# Data-Limited Stock Status Assessment of Bonga Shad, *Ethmalosa fimbriata* (Bowdich, 1825) and Lesser African Threadfin, *Galeoides decadactylus* (Bloch, 1795) in the Central Gulf of Guinea

**DOI:** 10.3390/biology15120978

**Published:** 2026-06-22

**Authors:** Edwin Egbe Atem, Richard Kindong, Collins Etah Ayuk, Mustapha Sly Bayon, David Mboglen, Siquan Tian

**Affiliations:** 1 College of Marine Living Resource Sciences and Management, Shanghai Ocean University, No. 999, Hucheng Ring Road, Pudong New Area, Shanghai 201306, China; atemedwin58@gmail.com (E.E.A.); msbayon85@gmail.com (M.S.B.); david1mboglen@gmail.com (D.M.); 2 Specialty of Fisheries and Aquaculture, Limbe Nautical Arts and Fisheries Institute (LINAFI), Limbe BP 485, Cameroon; etahayuk@yahoo.com; 3 Ministry of Livestock Fisheries and Animal Industries, Yaounde BP 441, Cameroon; 4 National Engineering Research Center for Oceanic Fisheries, Shanghai Ocean University, Shanghai 201306, China; 5 Ministry of Education Key Laboratory of Sustainable Exploitation of Oceanic Fisheries Resources, Shanghai Ocean University, Shanghai 201306, China; 6 Collaborative Innovation Center for Distant Water Fisheries, Shanghai 201306, China; 7 Department of Fisheries and Aquatic Resources Management, University of Buea, Buea BP 63, Cameroon; 8 Freetown Fisheries Research Centre, Sierra Leone Agricultural Research Institute, Freetown P.M.B. 1313, Sierra Leone; 9 Specialized Research Station on Marine Ecosystems, Institute of Agricultural Research for Development (IRAD), Kribi BP 219, Cameroon

**Keywords:** AMSY, CMSY++, BSM, *Ethmalosa fimbriata*, *Galeoides decadactylus*, Central Gulf of Guinea

## Abstract

This study presents a comprehensive data-limited stock assessment of bonga shad (*Ethmalosa fimbriata*) and lesser African threadfin (*Galeoides decadactylus*) in the Central Gulf of Guinea using complementary catch- and abundance-based approaches, including Abundance-based Maximum Sustainable Yield (AMSY), Catch-based Maximum Sustainable Yield ++ (CMSY++), and the Bayesian State-space Schaefer Model (BSM). These models were applied because they are suitable for evaluating stock status in data-limited fisheries using catch and abundance information. While AMSY primarily uses abundance information, CMSY++ integrates catch and productivity priors, whereas BSM incorporates state-space error structures to account for observation uncertainty. Catch time series (1990–2021) were extracted from Food and Agricultural Organization (FAO) FishstatJ accessed in 2023, with catch values for 2022–2023 cautiously extrapolated from recent trends due to the temporary absence of updated official statistics. Standardized and scaled relative abundance indices from Cameroonian and Nigerian EEZ were used to support model estimation and assess the stock status. For *Ethmalosa fimbriata*, the results from CMSY++ and BSM yielded an MSY estimate of 126 × 10^3^ t and 95.5 × 10^3^ t, respectively, while for *G. decadactylus*, MSY from CMSY++ and BSM were 9.1 × 10^3^ and 13.4 × 10^3^, respectively. Stock status indicators suggested the stock was fully exploited based on both AMSY (F/F_MSY_ = 0.83) and CMSY++ (F/F_MSY_ = 1.03) and overfished based on BSM (F/F_MSY_ = 1.77). For *G. decadactylus*, the analysis based on AMSY suggested an overfished stock state (F/F_MSY_ = 1.2), while under CMSY++ and BSM, the stock is fully exploited. The log scale CPUE was symmetrical within the expected bounds, and the posterior parameter distributions were constrained, indicating that the model passed the convergence test and had robust parameter estimates. The study recommends maintaining catches within MSY-based reference points as the total allowable catch (TAC) and emphasizes the need for improved data continuity, regional collaboration, and precautionary management for long-term sustainability of fisheries resources in the Central Gulf of Guinea.

## Simple Summary

The Central Gulf of Guinea is a large marine ecosystem with a huge biodiversity of aquatic species, including bonga shad (*Ethmalosa fimbriata*) and lesser African threadfin (*Galeoides decadactylus*). These fish stocks are a major component of the protein source and are important for the economy of coastal communities in the Central Gulf of Guinea. Application of the general stock assessment methods is a major setback due to limited catch and effort data. Therefore, this study applies the complementary catch-only and abundance-based approaches to determine the stock status of bonga shad and lesser African threadfin (LATF) for proper management of these fisheries in the Central Gulf of Guinea. Our findings showed that bonga shad is in good stock state but presents a risk of long-term stock depletion as the estimated MSY value was below the last year’s catch, whereas the MSY estimate for LATF was close to the last year’s catch, suggesting that LATF is near an overfished state. This research recommends species-specific and precautionary management measures for the sustainability of bonga shad and LATF fisheries in the Central Gulf of Guinea.

## 1. Introduction

Global fisheries continue to face intense pressure, with nearly one-third of assessed stocks now classified as overfished and many more showing signs of persistent declines in productivity [1,2]. This situation is particularly pronounced in tropical fisheries, especially in many West African countries bordering the Gulf of Guinea; incomplete or inconsistent time-series catch, effort, and age structure data remain a major constraint to stock assessment and sustainable management [3,4]. Limitations in long-term time-series data, abundance indices, and age-structured information often restrict the application of conventional analytical assessment methods. Consequently, only a small fraction of the world’s marine fisheries, estimated at roughly 12%, are effectively assessed and managed using conventional analytical approaches [2].

In the Central Gulf of Guinea (CGG), situated within the FAO Eastern Central Atlantic Area 34, small-scale fisheries dominate coastal exploitation, accounting for approximately 70–85% of total marine fish landings and providing employment, income, and food security to the majority of coastal households [5]. Two key species underpinning this sector are the bonga shad *Ethmalosa fimbriata* (Bowdich, 1825), a small pelagic clupeid widely distributed in West African estuaries and coastal shelves [6,7], and the lesser African threadfin *Galeoides decadactylus* (Bloch, 1795), a coastal demersal polynemid inhabiting sandy and muddy substrates of the Gulf of Guinea [8].

Bonga shad and lesser African threadfin (LATF) are essential species in the Gulf of Guinea, not only in terms of fisheries productivity but also for food security and livelihoods. Both species contribute substantially to artisanal fisheries production and play an important role in regional food security and livelihoods. In several coastal communities, a large proportion of households depend directly or indirectly on fisheries-related activities for income, nutrition, and employment [9]. Both species constitute a significant portion of the catch in Cameroon and Nigeria. Both species are also major sources of animal protein for coastal populations, about 40%% animal protein intake for coastal communities [10]. This high reliance on fish protein underscores the crucial role these species play in regional food security. The species are vital for the local economies of the coastal populations, yet comprehensive stock assessments remain scarce. According to the FAO/CECAF working group reports, catches of bonga shad increased from 30,883 t in 1990 to a peak of 152,805 t in 2019, while LATF catches rose from approximately 2560 t in 1990 to 11,282 t in 2018. Both species show declining trends in historical catches in recent years, with bonga shad decreasing from 152,805 t in 2019 to 149,041 t in 2021, approximately 2.5% decrease. For LATF, catches decreased from 11,282 t in 2018 to 10,244 t in 2021, about 9% decrease, as reflected in regional assessments and changes in catch patterns. In addition to fishing pressure, both species exhibit short life spans, strong environmental sensitivity, and wide spatial distributions, making them especially vulnerable to overexploitation under open-access conditions [11,12]. Given the lack of detailed age-structured data in the region, adopting reliable data-limited assessment approaches is essential for evaluating stock status, identifying long-term trends, and supporting precautionary fisheries management [13].

Data-limited assessment models have been developed to estimate biological reference points (BRPs) and evaluate stock status using available catch and auxiliary data [14,15,16]. Among these, AMSY, CMSY++, and BSM use catch time series, life-history traits, and limited abundance indices to estimate key reference points such as maximum sustainable yield (MSY). Comparing these models allows cross-validation of results, improved understanding of uncertainty, and more informed management decisions [17,18,19]. AMSY (Abundance-based MSY estimation) requires catch data, an abundance index (e.g., CPUE), and priors for intrinsic growth rate (r) and carrying capacity (K) and is particularly useful where reliable abundance proxies exist. CMSY++, an extension of the CMSY Monte Carlo approach [18], applies a modified Schaefer model and can assess exploitation status using catch-only data supplemented with productivity and depletion priors, optionally incorporating relative abundance indices. BSM (Bayesian Surplus Production Model) integrates catch and abundance indices within a state-space Bayesian framework, explicitly accounting for process and observation uncertainty to generate robust estimates of MSY, biomass trajectories, and exploitation rates. These complementary approaches were applied because they are suitable for evaluating stock status in data-limited fisheries using catch and abundance information.

Small pelagic bonga shad and demersal lesser African threadfin constitute major commercial fisheries in the CGG, primarily exploited by artisanal and semi-industrial fleets using purse seines and gillnets with mesh sizes of 30–42 mm and 40–60 mm, respectively, operated by vessels ranging from 5 to 15 m powered by 30–40 HP outboard engines [5,20]. These fisheries contribute approximately 80% of total marine fish landings in CGG countries [5,21]. Recent assessments in the region include European pilchard (*Sardina pilchardus*) in northern West African waters [22], Atlantic bonito (*Sarda sarda*) off the Senegalese EEZ [23], and LATF was assessed using length-frequency data within FAO FiSAT II software [24] and documented the vulnerability of this fish stock in Sierra Leonean waters. However, stock assessment efforts in the Central Gulf of Guinea remain limited due to the lack of high-resolution data, such as age-structured information, fishery-independent surveys, and consistent biological sampling, which are required for conventional stock assessment models. To address this limitation, the present study applies a combination of complementary data-limited models (AMSY, CMSY++, and BSM) using available catch and abundance index data to estimate biological reference points. The objective of this study was to estimate biological reference points and propose management advice for *Ethmalosa fimbriata* and *Galeoides decadactylus* in the Central Gulf of Guinea. To achieve this objective, we addressed the following research questions: (i) What are the estimates of maximum sustainable yield (MSY) and associated biological reference points? (ii) What are the historical trends in biomass and exploitation rates? and (iii) Are the stocks currently overfished or experiencing overfishing?

## 2. Materials and Methods

### 2.1. Sources of Data

#### 2.1.1. Study Area, Stock Definition, and Life History Parameters

##### Study Area

**Figure 1:**
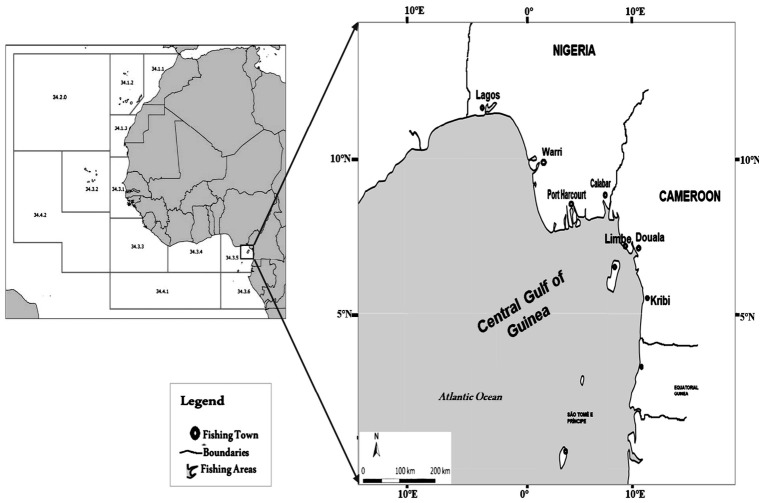
Major fishing areas in the Central Gulf of Guinea are within FAO Fishing Area 34. The map was adapted and modified from the FAO Major Fishing Area 34 map (Food and Agriculture Organization of the United Nations, https://www.fao.org/fishery/en/area/34; accessed on 12 March 2026).

The Central Gulf of Guinea (CGG) lies between latitudes 3° N–7 °N and longitudes 5° E–10° E. CGG is a subregion of FAO Major Fishing Area 34, including the coastal zones of Cameroonian and Nigerian EEZ (Figure 1), and the assessment is limited to these two countries due to data availability covering tropical coastal waters, highly productive for small-scale fisheries [25]. The commercial fisheries sector within CGG is broadly categorized as an artisanal, semi-industrial, and industrial fishery. The artisanal mechanized fishing boats measuring 6–15 m are powered by outboard engines, while semi-industrial and industrial trawlers are normally above 15 m long and powered by inboard engines.

##### Stock Definition

The fishery in FAO fishing Area 34 is divided into four (04) stocks by CECAF: Northern, Western, Central, and Southern stocks, and this study is focused on the central stocks, including bonga shad, *Ethmalosa fimbriata,* and lesser African threadfin, *Galeoides decadactylus*.

##### Life History Parameters

Life-history inputs for *Ethmalosa fimbriata* and *Galeoides decadactylus* were derived from length-frequency analyses. Growth parameters were estimated from length-frequency (LFQ) data collected monthly during 2024 from commercial landings in the region. Although the primary assessment period was based on catch data ending in 2023, the use of recent biological samples was considered appropriate. This is because growth and mortality parameters are generally assumed to remain relatively stable over short temporal scales in the absence of major environmental or ecological shifts. Individual fish lengths were grouped into 1 cm size classes (bin length), and a time-series LFQ matrix was constructed across sampling months for each species. The von Bertalanffy growth function (VBGF) parameters (L∞, K) were fitted using ELEFAN with simulated annealing (ELEFAN-SA) implemented in TropFishR. This method identifies modal progression in LFQ distributions across time. Natural mortality (M) was subsequently estimated using Ricker’s empirical relationship, which incorporates the VBGF parameters (L∞ and K) and the maximum observed length (Lmax). These analyses were conducted specifically for the present study using the available LFQ datasets and are summarized here to ensure reproducibility.

To account for biological uncertainty, parameters were incorporated as ranges or prior distributions rather than precise point estimates. These priors informed the estimation of intrinsic growth rate (r), depletion levels, and reference points within the CMSY++, AMSY, and BSM frameworks, and their uncertainty was propagated into the 95% confidence intervals of MSY and stock status indicators.

The natural mortal fir both species was calculated using the method described by [26]. All inputs, equations, and resulting life-history parameters are provided in Supplementary Table S1.

Their life-history traits imply relatively high population productivity and resilience to exploitation, which is consistent with the “medium-high resilience” classification commonly applied in data-limited stock assessment frameworks.

Although FAO/CECAF traditionally treats bonga shad and LATF in West Africa as a single shared stock, recent genetic studies suggest the presence of partially isolated sub-populations across its distribution range [25,27]. However, within the central Gulf of Guinea, gene flow appears relatively continuous, supporting the treatment of this region as a broadly connected production unit. Consequently, aggregating catch and effort data from the principal contributing countries is considered appropriate for data-limited stock assessment using CMSY/BSM approaches. Nevertheless, the resulting estimates of MSY and reference points should be interpreted as indicators of the productivity of a multi-country management unit rather than a strictly discrete biological stock.

#### 2.1.2. Catch and Relative Abundance Indices Data Processing

Annual catch time-series data for Bonga shad and LATF (1990–2021) were obtained from the official Food and Agriculture Organization/Fishery Committee for the Eastern Central Atlantic (FAO/CECAF) capture production database, accessible online via FAO FishStatJ statistical query system [28], and remain the most comprehensive source available in this region. The catch data were considered in tons (t) and aggregated across the Cameroonian and Nigerian EEZs to approximate a biologically coherent regional stock because they are highly migratory species with broad distribution across the region. Within FAO Major Fishing Area 34, CECAF conventionally subdivides coastal resources into four broad assessment units (Northern, Western, Central, and Southern stocks). The present study focuses on the Central Gulf of Guinea stock, where both species are widely distributed and intensively exploited.

Catch statistics from Cameroon and Nigeria were retained for analysis because these two countries account for the majority of reported landings and provide the most continuous and complete time series within the central region. Records from neighboring countries were comparatively sparse or discontinuous and were therefore excluded to avoid introducing gaps and instability in surplus production model estimation.

For the purposes of CMSY/BSM analyses, the resulting dataset represents a regional multi-country production unit rather than a strictly discrete biological stock; therefore, estimated reference points (MSY) should be interpreted as regional indicators of exploitation status.

**Figure 2:**
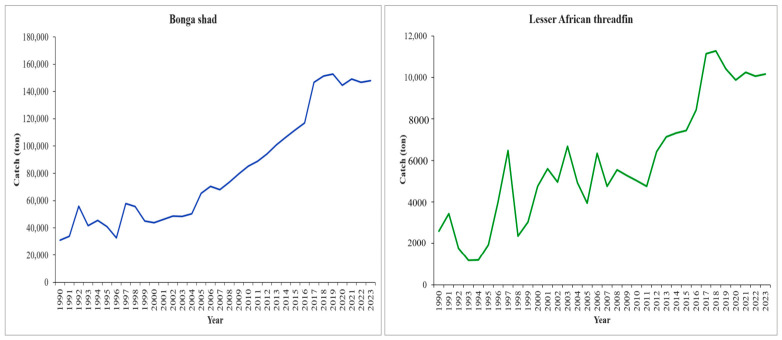
Catches of bonga shad and lesser African threadfin recorded from 1990 to 2023 off the central Gulf of Guinea.

Catch values for 2022 and 2023 were cautiously extrapolated from recent trends using the mean of the last three observed years (2019, 2020, and 2021) to extend the catch data from 1990 to 2023 (Figure 2) due to the temporary absence of updated official statistics. This conservative trend-neutral approach avoids introducing artificial increases or declines that may arise from linear extrapolation and is commonly applied in data-limited assessments [16] and supports consistent model inputs. Bonga shad is a highly migratory small pelagic species with a broad distribution across FAO Area 34. Catches from Cameroon and Nigeria were aggregated to approximate a biologically coherent regional stock, consistent with previous CECAF working group assessments. The complete time series catch data used as input are provided in ST2(A) and ST2(B) for bonga shad and LATF, respectively.

Annual nominal catch per unit effort (CPUE) data for bonga shad were obtained from the Food and Agriculture Organization of the United Nations/Fishery Committee for the Eastern Central Atlantic FAO/CECAF working group report [25], while annual nominal CPUE data for LATF were obtained from FAO/CECAF working group reports [27]. For bonga shad, a single standardized relative abundance index derived from the Cameroonian fleet was available for the period 1994–2008. Although the catch time series extends to 2023, no abundance index was available for the more recent years. In the absence of index data, the model relies on the observed catch trajectory and prior assumptions to project biomass dynamics beyond 2008. While this does not prevent model convergence, it reduces the strength of data-driven inference in the terminal years and increases uncertainty in recent stock status estimates. This limitation is inherent to data-limited assessments and is explicitly considered when interpreting model outputs.

**Figure 3:**
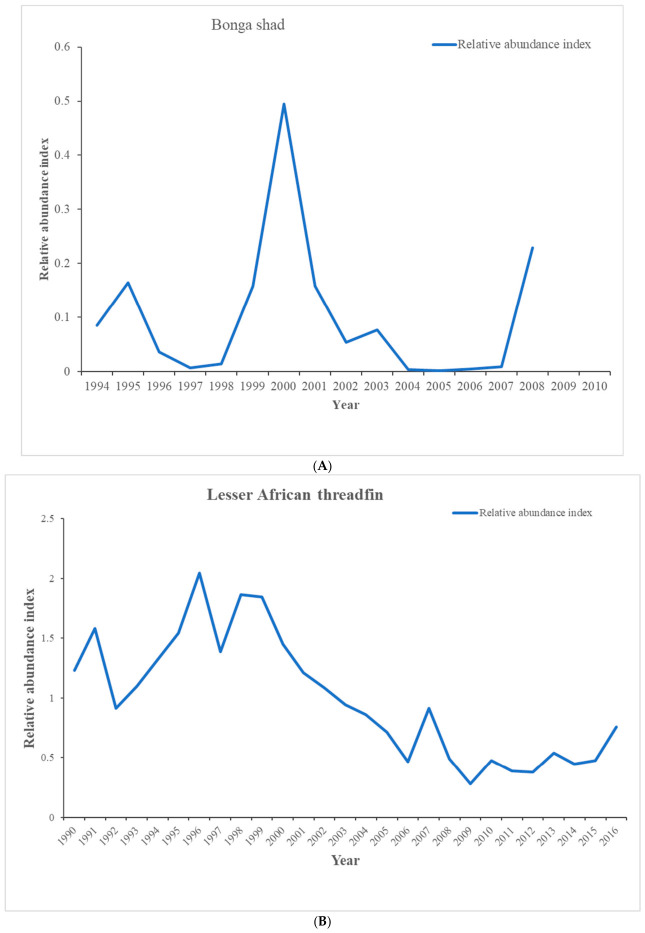
(**A**). Time series standardized and scaled relative abundance index for bonga shad from 1994 to 2008 off the CGG. (**B**). Time series standardized and scaled relative abundance index for lesser African threadfin (LATF) from 1990 to 2016 off the CGG.

However, because nominal CPUE values may be influenced by changes in fishing power, spatial coverage, and sampling practices, they were not used directly. Standardized abundant indices for *E. fimbriata* (Supplementary Table S3a) and *G. decadactylus* (Supplementary Table S4a) were derived from a previously validated spatiotemporal standardization analysis [29]. The CPUE values for both species were standardized to remove temporal variability associated with changes in fishing effort and improve comparability across years. The resulting standardized time series abundant indices are not raw catch rates and do not retain physical effort units (kg·day^−1^ or kg·vessel^−1^). For bonga shad, a single reliable standardized index was available from the Cameroonian fleet and spanned from 1994 to 2008. For numerical stability and comparability within the surplus production models, the index was scaled by its temporal mean to obtain a dimensionless relative abundance series (Figure 3A).

For LATF, four standardized abundant indices were available, derived independently from Cameroonian and Nigerian fleets using different effort metrics and spanning from 1990 to 2016. Because these indices differ in magnitude due to fleet-specific scaling, each series was first scaled by its temporal mean and then averaged annually to produce a single composite regional abundance index to ensure comparability across fleets with different effort units and catchability for this species (Figure 3B).

This transformation produced a dimensionless standardized relative abundance index, denoted as $I_{t}$, which preserves temporal trends while removing differences in magnitude among fleets.

The relationship between the relative abundance index and biomass is defined as:

$$I_{t}=q^{*}\cdot B_{t}$$

where

$I_{t}$ is the standardized relative abundance index at time t,
$B_{t}$ is biomass, and
$q^{*}$ is an effective catchability coefficient that absorbs the scaling factor introduced during standardization.

Under this formulation, catch data determine the absolute biomass scale, while the relative abundance index constrains the temporal trajectory of biomass through the estimation of $q^{*}$.

This principle is consistently applied across the AMSY, CMSY++, and Bayesian state-space biomass (BSM) models, while the standardized relative abundance index informs relative changes in stock size over time. Only years with satisfactory model diagnostics and consistent effort reporting were retained to minimize uncertainty. The complete standardized and scaled time series used as inputs to AMSY, CMSY++, and BSM are provided in Supplementary Tables S3b and S4b for bonga shad and LATF, respectively.

#### 2.1.3. Life-History Parameters for Model Priors

Life-history characteristics were compiled to ensure that the AMSY/CMSY parameterization was biologically defensible.

For bonga shad, the species is a short-lived, fast-growing clupeid characterized by early maturation (≈1 year), small maximum size, high natural mortality, and strong interannual recruitment variability typical of small pelagic fishes in the Gulf of Guinea. Such traits indicate high productivity and rapid population turnover. Accordingly, the stock was classified as high resilience, and a broad prior for intrinsic growth rate (r) consistent with small pelagic fishes was applied in CMSY/AMSY.

For LATF, growth is moderate with later maturation and longer lifespan relative to clupeids, suggesting intermediate productivity. The stock was therefore assigned medium resilience and a correspondingly narrower r prior. Recruitment variability in both species is influenced by seasonal upwelling, river discharge, and coastal environmental fluctuations characteristic of the Gulf of Guinea, which are known to drive cohort strength in West African coastal fisheries.

Because fishery-independent surveys and age-structured data are unavailable in the region, surplus-production models that rely on catch and relative abundance indices represent the most appropriate assessment framework. However, life-history information was explicitly used to constrain r–K priors and ensure biological realism of model outputs.

### 2.2. Modeling

#### 2.2.1. AMSY Model

AMSY is a new data-limited assessment method that estimates key fishery reference points, including Fmsy, F/Fmsy, and B/Bmsy, using time-series data of abundance indices (CPUE) when no catch data are available [30]. The standardized and scaled relative abundance index for bonga shad and LATF were incorporated directly into the likelihood of the AMSY models as biomass proxies. The model requires a prior for relative stock size (B/k, ranging from 0 to 1) in a certain year of the time series. AMSY uses this information and tests a high number of combinations of intrinsic growth rate (r) and carrying (K) and retains those that are compatible with the observed abundance trends and specified priors.

#### 2.2.2. CMSY++ and BSM

The CMSY ++ and Bayesian Schaefer Model (BSM) packages were used to estimate the key BRPs MSY, biomass relative to MSY (B/B_MSY_), and fishing mortality relative to MSY (F/F_MSY_), using time series data on catch, CPUE, and resilience. Estimates of productivity in surplus production models are derived from catch and abundance indices as described in the Fox and Schaefer surplus production models [30,31]. The CMSY++ is an optimization of CMSY. It uses data on catch and productivity with the application of an artificial neural network (ANN) to estimate initial and final stock status to give more reliable biomass priors. Additionally, CMSY++ adopts a multivariate log normal (MVLN) distribution to ascertain the negative correlation between *r* and *k* priors, resulting in a more realistic biomass estimate [30]. The Bayesian state-space implementation of the Schaefer production model (BSM) [32] uses catch and CPUE data and was incorporated for the assessment.

The population dynamics for bonga shad and LATF are described following CMSY++ based on the first derivative of the logistic growth curve according to [33]. and replacing their individual counts with their respective weights [17,31,34] below:

(1) $$B_{t+1}=B_{t}+r\left.{.B}_{t}\left(1-\frac{B_{t}}{k}\right.\right)-C_{t}+\epsilon_{t}$$

where

*B_t_*_+1_ is the exploited biomass in the year (*t +* 1), the existing biomass and catch in year t were, respectively, *B_t_* and *C_t_*, *k* is the carrying capacity, r is the intrinsic population growth rate, and $\epsilon_{t}$ is the observation error with $\epsilon_{t}∼N(0,\sigma_{process}^{2})$.

The following equation was applied considering that CPUE (index of abundance) was available:

(2) $$log{(I}_{t})=log\left.\left(q.\right.B_{t}\right)+\eta_{t},\eta_{t}∼N(0,\sigma_{obs}^{2})$$

where

$I_{t}$ = CPUE;
q = catchability coefficient;
$\eta_{t}$ = observation error.
Priors for r and k in CMSY++ are
r∼LogUniform($r_{mint,}r_{max}$);
k∼LogUniform($k_{mint,}k_{max}$).

#### 2.2.3. Stock Status and Estimation of BRPs for Bonga Shad and LATF

Stock status was estimated using the CMSY++ framework, which integrates the Catch-MSY method and a Bayesian Schaefer Model (BSM). Both approaches were run simultaneously to improve the robustness of the estimated parameters. Biological prior ranges were defined using resilience classifications in [16,35]. CMSY/AMYS rely primarily on catch time series and biologically informed priors for intrinsic growth rate (r) and relative biomass (B/k). The resilience value for Bonga shad and LATF was assumed to be “high” and “Medium”, respectively, and r prior range of 0.15–0.34 and 0.32–0.73 (Table 1) was used for Bonga shad and LATF, respectively, as the input prior. Priors were selected based on species resilience, exploitation history, and observed catch trajectories following CMSY++ guidelines.

The starting biomass relative to carrying capacity (*B/k*) for both species was assumed to fall between 0.2 and 0.8, reflecting moderate depletion at the start of the time series (1990). Final biomass (endb.low, endb.hi) and intermediate biomass (intb.low, intb.hi) priors were left unspecified (NA) due to limited information on depletion levels in those periods [36].

The same prior ranges and time series data were assigned in the BSM component; the available catch-per-unit-effort (CPUE) indices were additionally incorporated. This allowed the model to fit a production curve constrained by both catch data and relative abundance trends.

The probabilistic biomass ranges automatically estimate the relative biomass at the start and end of the time series data using the Markov Chain Monte Carlo method based on the anticipated level of depletion.

The carrying capacity ranges were calculated using the species’ maximum catch and resilience, following the Equations:

(3) $$k_{low}=\frac{max(Catch)}{r_{high}}\;\mathrm{and}\;k_{high}=\frac{4max(Catch)}{r_{low}}$$

(4) $$k_{low}=\frac{2max(Catch)}{r_{high}}\;\mathrm{and}\;k_{high}=\frac{12max(Catch)}{r_{low}}$$

where k_low_ and k_high_ are the lower and upper bounds of k.

Equation (3) accounts for the stocks with low prior biomass at the end of the given time series dataset, while Equation (4) is used for the stocks with high prior biomass.

The equation below connects the *CPUE* (relative abundance index) to the stock biomass (*B*) through the catchability coefficient (*q*):

(5) $${CPUE}_{t}={qB}_{t}$$

When r and k are calculated,

*MSY* = $\frac{rk}{4}$, or log(k) = log(4 MSY) + (−1) log(r);
$F_{MSY}$ = 0.5 *r* (fishing mortality corresponding to MSY), while
$B_{MSY}$ = 0.5 *k,* following the approach documented by [37] and the biomass below which recruitment is half of B_MSY_ as reported by several authors, including [38,39,40].

Table 2 shows the input values (relative biomass, *r*, *k,* and *q* ranges) for CMSY according to the work of several authors, including [32,41].

### 2.3. Data Analysis

The data were analyzed using the R codes for AMSY, CMSY++/BSM. The Markov chain Monte Carlo (MCMC) method [42] was employed, and the parameter estimation followed the Monte Carlo filtering framework implemented in CMSY++ rather than a classical multi-chain MCMC algorithm. A total of 50,000 candidate r-K-q parameter combinations were randomly sampled from prior ranges, and each combination was tested over 30 stochastic trials with process error (σr = 0.05–0.15) and CPUE observation error (σCPUE = 0.3). Combinations that produced biologically feasible biomass trajectories consistent with catch and CPUE data were retained, while non-viable solutions were discarded. At least 20 viable parameter sets were required for acceptance (maximum 5000 retained). Convergence and stability were evaluated by ensuring that posterior distributions of key parameters (r, K, MSY, B/BMSY, F/FMSY) were consistent across repeated runs. The AMSY model randomly selects *r* and *K*q pairs of the prior distribution to estimate the catch that can match the abundance, together with the two prior information inputs. Additionally, a series of filters is used to eliminate any *r*-*K*q combinations that produce negative catches or do not meet the actual exploitation value. Finally, the ‘viable’ *r*-*K*q pairs are used to calculate MSY*q*. The AMSY model requires time-series CPUE data, prior distribution range for *r*, and prior information on the relative population abundance level (*Bt*/*K*).

The prior distribution range for the population parameter *r* for both species was assigned based on the resilience grading table from Fishbase [43].

### 2.4. Model Fit and Retrospective Analysis

Residual diagnostics were used to evaluate the goodness-of-fit of AMSY, CMSY++, and BSMs. Model performance was assessed by examining standardized residuals of log-transformed CPUE against time to verify: (i) random scatter around zero with no systematic trend, (ii) homoscedastic variance, (iii) absence of strong autocorrelation (lag-1 autocorrelation, and (iv) lack of persistent runs of positive or negative residuals. In addition, only runs with stable parameter convergence and biologically plausible r–k combinations were retained. Based on the CMSY/BSM graphical diagnostics, panels flagged in green indicated that residual patterns met these criteria, whereas red panels indicated violations, and those runs [18] were rejected.

Retrospective analysis was formally conducted using the AMSY model by sequentially removing recent years (number of years *n* = 4) of the abundance index time series data and refitting the model to evaluate the stability of biomass and exploitation estimates. CMSY++ does not implement a formal retrospective analysis because it operates in equilibrium r-k space. However, to assess the robustness of the last-year stock status estimates from the Bayesian Schaefer Model (BSM) component within the CMSY++ framework, a truncated-based sensitivity analysis was done by sequentially shortening the catch and CPUE time series data.

## 3. Results

### 3.1. Stock Status Based on AMSY, CMSY++, and BSM for Bonga Shad, Ethmalosa fimbriata

According to the assessment results for bonga shad from AMSY, CMSY++, and BSM, the models generate two analyses based on viable r-k pairs and stock status management results. The analyses from the three models show that the logr-k space explored and the r-k pairs in dark gray were consistent with the catches and prior information in all three models (Supplementary Figure S1).

**Figure 4:**
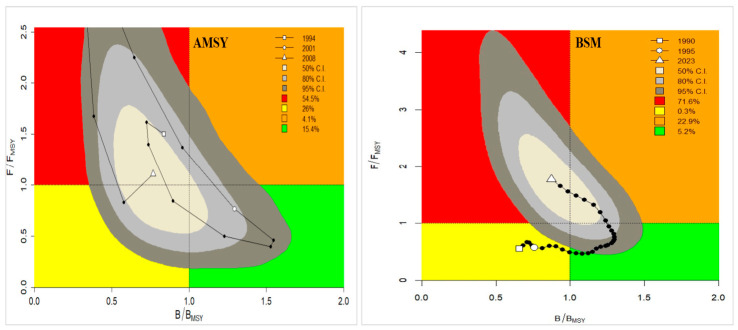
Kobe plots showing the state of exploitation and relative biomass for bonga shad based on AMSY and BSMs in the CGG.

The three models produced differing estimates of the intrinsic growth rate (r), with AMSY yielding a higher value (r = 0.35 yr^−1^) compared to CMSY++ (r = 0.17 yr^−1^) and BSM (r ≈ 0.21 yr^−1^). This discrepancy reflects differences in data inputs and model structure. AMSY relied primarily on the shorter abundance index covering 1994–2008, a period characterized by relatively high interannual variability and declining abundance trends. Consequently, the model was more sensitive to fluctuations within this limited time series compared to CMSY++ and BSM, which incorporated longer catch histories and additional sources of information. In contrast, CMSY++ and BSM are more strongly constrained by the full catch time series extending to 2023, resulting in comparatively lower and more conservative estimates of productivity. Considering the life-history characteristics of *E. fimbriata* as a small pelagic species, the CMSY++ and BSM estimates may represent more biologically plausible central tendencies, while the AMSY estimate reflects the upper bound of plausible productivity, suggesting that biomass was close to the limit reference point at the end of the abundance index period (2008). However, the assessment extends to 2023 based on catch data, and all stock status indicators reported in this study correspond to the terminal year of the assessment (2023). The estimated MSY of bonga shad from the CMSY++ and BSM were 126 (CI: 90.3–201) × 10^3^ t and 95.5 (CI: 73.5–134) × 10^3^ t, respectively (Table 3). The values of MSY from both methods were above the average catch value (81.729 × 10^3^ t) and very close to the catches from 2013 towards the later years. The last year (2023) fishing mortality from BSM (F_2023_ = 0.19) was higher than F_MSY_ = 0.10. Furthermore, the last year’s biomass (B_2023_/B_MSY_ = 792 × 10^3^ t) from the BSM was lower than the B_MSY_ (915 × 10^3^ t). The assessment results for bonga shad from the three models are found in Supplementary Material Table S5a. The Kobe management plots for the state of exploitation of bonga shad (Figure 4) based on AMSY and CMSY indicated that bonga shad is fully exploited, AMSY (F_2008_/F_MSY_ = 0.81 and B/B_MSY_ = 0.76) and CMSY++ (F_2008_/F_MSY_ = 1.03 and B/B_MSY_ = 1.08), suggesting biomass is close to the limit reference point in the last year (2008). However, the Bayesian Schaefer Model, which incorporates both catch and abundance information, suggests that current fishing mortality exceeds F_MSY_ (F_2008_/F_MSY_ = 1.77) and biomass is slightly below B_MSY_ (B_2008_/B_MSY_ = 0.87), indicating that the stock in the Central Gulf of Guinea could be experiencing overfishing. Considering that BSM incorporates uncertainty and provides more robust state-space estimates, the overall weight of evidence supports the conclusion that the stock is currently overfished in the CGG.

#### Stock Status Based on AMSY, CMSY++, and BSM for Lesser African Thread (LATF), *Galeoides decadactylus*

The assessment results for LATF from AMSY, CMSY++, and BSM showed that the r-k space explored, and the viable r-k pairs (dark gray) were consistent with the observed catch trajectories and the specified prior information, indicating a broad consistency among the three assessment approaches (Supplementary Figure S2).

The estimated biological reference points and stock status for lesser African thread (LATF), based on AMSY, CMSY++, and BSMs, are summarized in Table 4.

All three models produced relatively high intrinsic growth rates, with AMSY (r = 0.49 yr^−1^), CMSY++ (r = 0.33 yr^−1^), and BSM (r ≈ 0.356 yr^−1^) indicating moderate-high productivity and rapid rebuilding potential under reduced fishing pressure. Maximum sustainable yield estimates were 9.1 × 10^3^ t yr^−1^ (95% CI: 6.7–13.2) from CMSY++ and 13.4 × 10^3^ t yr^−1^ (95% CI: 9.2–20.4) from BSM, suggesting comparable stock productivity across models. Stock status indicators showed some variation among approaches. AMSY suggested lower biomass (B/B_MSY_ = 0.50) and fishing mortality above F_MSY_ (F/F_MSY_ = 1.20), implying depletion. In contrast, the CPUE-informed models indicated healthier conditions as CMSY++ estimated biomass slightly above the MSY target (B/B_MSY_ = 1.08) with fishing close to Fmsy (F/F_MSY_ = 1.05), while BSM estimated biomass near B_MSY_ (B/B_MSY_ = 0.91) and fishing below F_MSY_ (F/F_MSY_ = 0.85). The detail assessment results for LATF from the three models are documented in Supplementary Table S5b.

**Figure 5:**
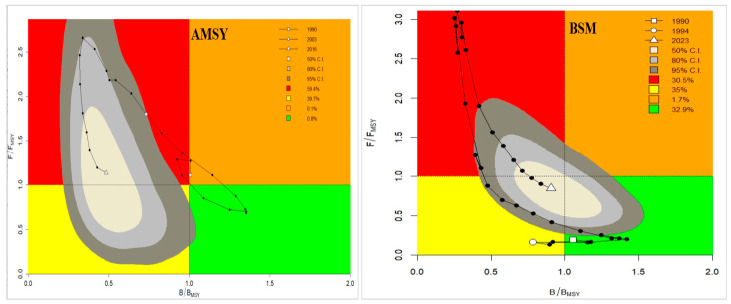
Kobe management plots showing the state of exploitation and relative biomass for LATF based on AMSY and BSMs in the CGG.

The Kobe plots (Figure 5) corroborated these findings, showing recent years concentrated in the yellow-green quadrants, suggesting recovery from earlier depletion and current exploitation near sustainable levels. Considering the higher reliability of the relatively abundant index-integrated BSM results, the LATF stock in the Central Gulf of Guinea is therefore best characterized as fully exploited but not currently overfished.

### 3.2. Model Fitting and Retrospective Analysis

**Figure 6:**
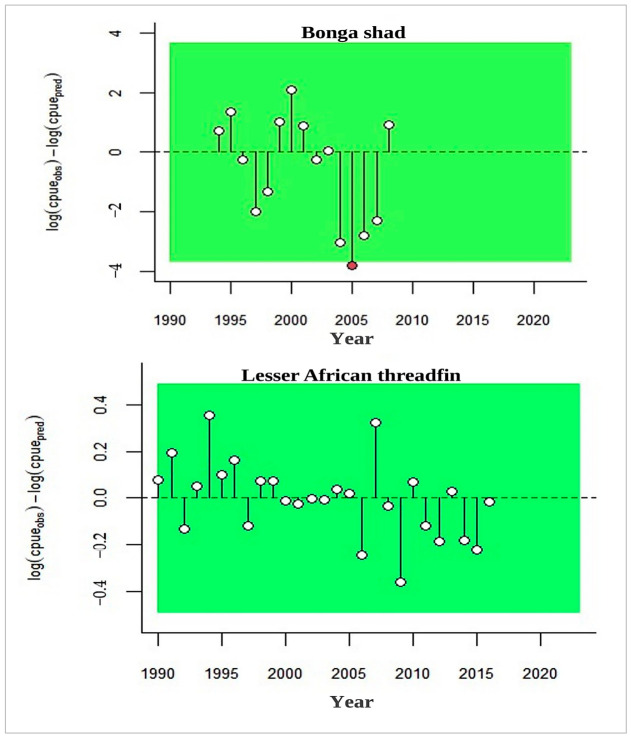
Log-CPUE residuals diagnostic plots for Bonga shad and lesser African threadfin from the BSMs framework.

The residual diagnostics indicate an overall satisfactory model fit for both *Ethmalosa fimbriata* and *Galeoides decadactylus* (Figure 6).

The log-scale CPUE residual diagnostics indicated satisfactory model performance for both stocks. Residuals were randomly distributed around zero with no clear temporal trends or systematic bias, suggesting that the BSM adequately captured interannual biomass dynamics. For Bonga shad, most residuals fell within ±2 log units, with a single pronounced negative outlier around the mid-2000s, which reflects anomalous effort concentration or reporting inconsistencies rather than persistent model bias. Residuals for lesser African threadfin were smaller in magnitude and more tightly clustered around zero, indicating an excellent fit and strong internal consistency between observed and predicted standardized relative abundance index. The standardized relative abundance index exhibited a declining trend despite increasing catches, resulting in a significant negative correlation between the two series. This suggests that the index may not reliably track underlying biomass dynamics.

Model outputs incorporating CPUE showed constrained parameter estimates and reduced variability, whereas catch-only models produced broader and more internally consistent posterior distributions. Consequently, catch-only results are considered more reliable for interpreting stock status.

Overall, the absence of systematic patterns supports the reliability of the BSM estimates. The posterior parameter distributions derived from BSM and CMSY++ for bonga shad (Supplementary Figure S3) and LATF (Supplementary Figure S4) were symmetrical and confined within a biologically plausible interval, indicating good model convergence and robust parameter estimation. Quantitative diagnostics, including residual standard deviations and autocorrelation checks, confirmed a satisfactory model fit for both *E. fimbriata* and *G. decadactylus*. The residuals were randomly distributed with no significant autocorrelation, as supported by the residual standard deviation values and runs test results.

**Figure 7:**
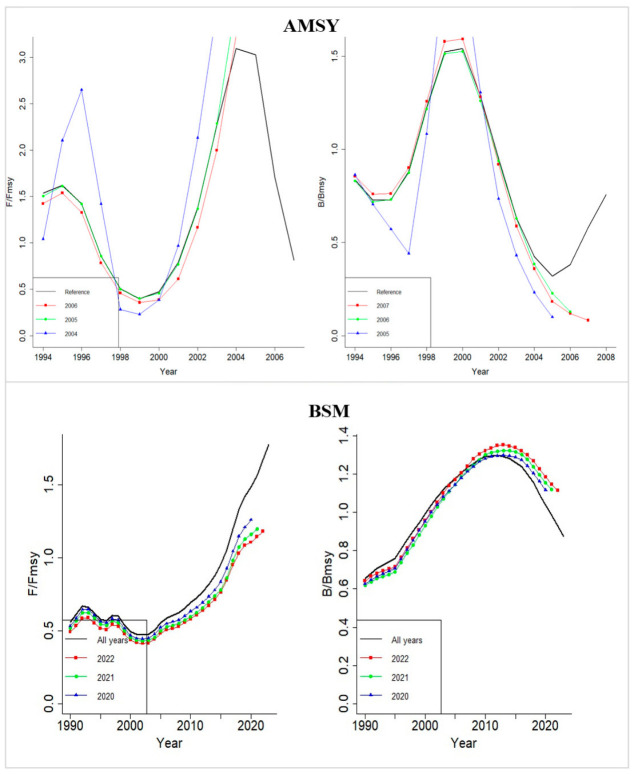
Retrospective plots for bonga shad from AMSY and BSMs in the CGG.

The three-year retrospective analysis for Bonga shad (Figure 7) revealed generally consistent trends across assessment models. For AMSY, the truncated runs (2004–2006 peels) closely tracked the reference trajectory over most of the time series and showed minimal deviations in the terminal years. Earlier truncations tended to modestly overestimate recent fishing pressure, indicating a small terminal-year effect. Nevertheless, all peels reproduced similar relative biomass trajectories, including the late-1990s increase followed by a gradual increase after 2001, suggesting relatively small deviations between peeled and full model trajectories, suggesting limited retrospective inconsistency.

Retrospective diagnostics from the BSM within the CMSY++ framework showed stronger stability and tighter overlap among truncated runs, with no clear systematic bias in either exploitation rate or relative biomass. This consistency indicates that the BSM provides more robust and internally coherent estimates for Bonga shad in the study region.

**Figure 8:**
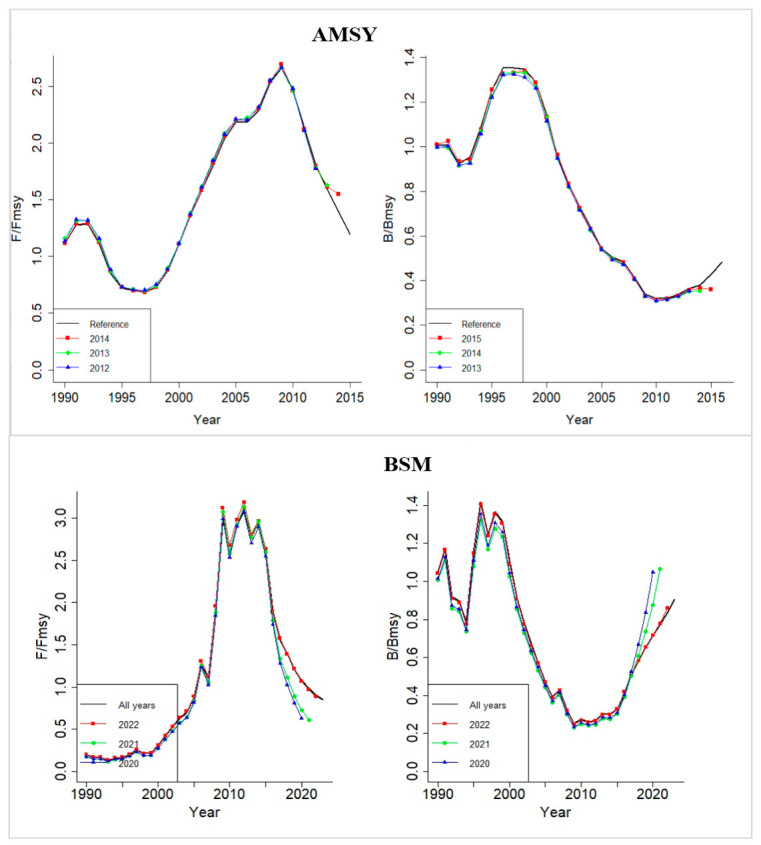
Retrospective plots for lesser African threadfin from AMSY and BSMs in the CGG.

The retrospective analysis for lesser African threadfin (LATF) (Figure 8) shows coherent but model-dependent patterns across all assessment models. For AMSY, successive retrospective peels indicate that earlier terminal-year runs tended to underestimate fishing mortality (F/F_MSY_) and overestimate biomass status (B/B_MSY_). As additional years of data were incorporated, F/F_MSY_ was consistently revised upward while B/B_MSY_ was revised downward, revealing a systematic retrospective bias and suggesting that AMSY is sensitive to recent information.

In contrast, the Bayesian Schaefer Model (BSM) exhibited stronger convergence among retrospective peels, particularly for biomass trajectories. Terminal-year biomass estimates showed only minor upward adjustments, and fishing mortality was slightly revised downward as new data were added. The absence of strong directional shifts and the tighter clustering of trajectories indicate improved stability and reduced retrospective bias. Overall, these diagnostics suggest that the explicit estimation of biomass dynamics and process error in BSM provides more internally consistent and robust estimates than AMSY for this stock.

## 4. Discussions

The Central Gulf of Guinea is located in FAO area 34 in West Africa and supports highly productive small pelagic and coastal demersal fisheries contributing to food security, employment, and sustainable livelihoods in coastal West African countries [44]. However, these fisheries are challenged by increasing fishing pressure and limited management measures, leading to overexploitation of marine resources. Refs. [45,46] pinpointed the prevalence of “hidden” overfishing in tropical fisheries, a pattern later confirmed through regional catch reconstructions and stock assessments in the West African coastline [47,48,49]. Given that fishing pressure on most small pelagic and demersal species in coastal waters continues to rise, it is essential to evaluate stock status and inform precautionary management decisions using the widely applied data-limited approaches [50,51]. MSY-based biological reference points, particularly B/B_MSY_ and F/F_MSY,_ are a popular framework adopted to ascertain the status of fisheries [52,53,54]. The current study applies for the first time three complementary stock assessment approaches in the Central Gulf of Guinea: an abundance-index-based method (AMSY), a catch-based Bayesian biomass reconstruction model (CMSY++), and a data-moderate integrated catch-CPUE model (Bayesian Schaefer Model, BSM) to estimate MSY and stock status of bonga shad and lesser African threadfin. The combined use of these models allows cross-validation of results across different data assumptions and improves the robustness of management inference for the sustainability of these resources.

This study estimated the stock status of *Ethmalosa fimbriata* and *Galeoides decadactylus* in the Central Gulf of Guinea using AMSY, CMSY++, and Bayesian Schaefer Model (BSM) models.

The comparison between catch-only and catch-plus-index models revealed inconsistencies in the CPUE time series for bonga shad. AMSY relies heavily on abundance indices; CMSY++ depends strongly on catch and resilience priors; BSM incorporates state-space structure and handles observation error differently; different priors and observation assumptions lead to different reference points. Convergence among models strengthens confidence, while divergence reflects uncertainty inherent in data-limited fisheries. Specifically, increasing catch trends were not reflected in the standardized abundance index, resulting in structural tension within the models. This led to unrealistic parameter behavior, including extremely low catchability coefficients and compressed carrying capacity estimates.

Such patterns are indicative of a non-informative or biased abundance index, which may arise from unaccounted changes in fishing effort, gear efficiency, or spatial targeting. Consequently, the inclusion of CPUE in this case may distort stock status estimates rather than improve them.

The models incorporating abundance indices may better capture temporal stock dynamics, state-space frameworks may reduce observation noise; outputs consistent with known fishery history and biological characteristics were considered more robust. Therefore, greater emphasis is placed on catch-only model outputs, which provide stable and internally consistent estimates and are informative under the available data conditions.

The stock parameter estimates for *E. fimbriata* from AMSY and CMSY++ showed that the stock is fully exploited, while BSM indicated the stock is overfished. The estimated intrinsic growth rate (r) for *E. fimbriata* differed across models, with AMSY providing the highest value (0.35 yr^−1^), followed by BSM (0.21 yr^−1^) and CMSY++ (0.17 yr^−1^). The AMSY estimate exceeded those of CMSY++ and BSM by approximately 51% and 40%, respectively, highlighting moderate variability among modeling approaches. These variations reflect different assumptions regarding the species’ productivity. Similarly, the estimated carrying capacities (*K*) differed by 36.3% between CMSY++ and BSM, with CMSY++ indicating a higher potential biomass. Both approaches estimated comparable intrinsic growth rates and carrying capacities, indicating a stock with moderate productivity, consistent with the life-history traits of small pelagic clupeids. The close agreement in MSY estimates between CMSY++ (126 × 10^3^ t; 95% CI: 89–195 t) and BSM (95.3 × 10^3^ t; 95% CI: 73.5–132 t) further underscores the robustness of the models. Recent catches (2019–2023) ranged from 144,580 to 152,805 t and remained consistently above the estimated MSY. This suggests that the stock is being harvested at levels exceeding sustainable limits. Despite uncertainties in reporting, reducing fishing pressure will be necessary to bring exploitation in line with biological reference points and ensure long-term sustainability. The exploitation rate in the terminal year exceeds sustainable levels under the BSM and borderline in CMSY++ and AMSY. These results are similar to those of [55], who previously reported the stock of *E. fimbriata* to be slightly overexploited in Gambian waters. Such concordance could be attributed to limited data on species, as documented by [56,57]. Therefore, the apparent increase in exploitation is more plausibly attributed to data misreporting rather than confirmed biological changes in stock status.

A degree of uncertainty remains inherent in the present assessment due to the data-limited nature of the fisheries and the assumptions underlying the applied models. Potential uncertainties may arise from inaccuracies in historical catch statistics, which may obscure differences in fishing efficiency and exploitation patterns over time. In addition, Uncertainty is also associated with the selection of prior ranges for key parameters such as intrinsic growth rate (r), carrying capacity (k), and depletion levels (B/k), particularly under limited biological information. Furthermore, differences observed among AMSY, CMSY++, and BSM outputs reflect structural uncertainty associated with model assumptions, data treatment, and error structures. The analyses also assume a single-stock dynamic for each species, although potential stock mixing and environmental variability within the Gulf of Guinea ecosystem may influence population dynamics and recruitment processes. Consequently, the results should be interpreted within a precautionary management framework. Nevertheless, the general convergence of model outputs toward similar stock status trends increases confidence in the overall conclusions and supports the need for strengthened fisheries management and continuous monitoring programs.

Regarding the stock parameter estimates for *G. decadactylus*, CMSY++ (MSY = 9.1 × 10^3^ t·yr^−1^) and BSM (MSY = 13.4 × 10^3^ t·yr^−1^) produced comparable MSY estimates, with an overall range of 9.1–13.4 × 10^3^ t·yr^−1^ and substantially overlapping 95% confidence intervals. Comparison with recent catch statistics indicates that the mean annual landings during 2019–2023 were consistently close to this MSY range, suggesting that exploitation is near the stock’s sustainable production capacity. This convergence indicates strong consistency between the production-based CMSY++ and the state-space biomass BSM approaches. Similar agreement between CMSY and BSM has been observed by [16], who reported that parameter estimates for 25 out of 28 assessed data-limited stocks were not significantly different. Furthermore, Ref. [16] demonstrated that both methods can yield comparable and robust MSY estimates when reliable catch and abundance information are available. Ref. [58] carried out a multi-stock assessment in West Africa, using CMSY++ and BSM, and reported similar results with overlapping uncertainty bounds. AMSY suggests the stock is overfished, while both CMSY++ and BSM indicate the stock is fully exploited. Divergence in AMSY reflects its sensitivity to recent CPUE increases, whereas BSM integrates biomass dynamics and fishing mortality more realistically, as documented by [59]. The strong negative correlation observed between catch and CPUE for LATF indicates a lack of coherence between the two datasets. This inconsistency may arise from unaccounted changes in fishing effort, spatial targeting, or limitations in the CPUE standardization process. This is also influenced by the hyperstability of CPUE, which may remain stable even when biomass declines because: fish aggregate spatially, fishers target dense schools, and improved fishing efficiency masks stock depletion. Increasing vessel efficiency can distort CPUE trends. Combining artisanal and industrial fleets may obscure actual abundance patterns. However, CPUE may not always remain directly proportional to stock abundance in data-limited fisheries because of factors such as hyperstability, technological improvements, and spatial aggregation of fish schools, potentially affecting the reliability of abundance indices used in the models.

When such inconsistencies are present, the inclusion of CPUE in surplus production models can lead to biased parameter estimates, as the model attempts to reconcile conflicting signals. This was evident in the present study, where CPUE-based models produced compressed parameter ranges and divergent stock status indicators.

In contrast, catch-only models avoid this structural conflict and provide more stable estimates under data-limited conditions. Therefore, greater confidence is placed in catch-only results for LATF.

The estimated MSY for bonga shad from CMSY++ and BSMs was below the last year catch (147.9 × 10^3^ t·yr^−1^), while the estimated MSY for LATF from the CMSY++ model was close to the last year catch, and BSM was well above the last year catch (10.2 × 10^3^ t·yr^−1^).

The results of [60] revealed that, when the MSY is closer to the last year catch, the stock is considered to be fully exploited [59,61]. Bonga shad is transitioning from a fully exploited condition towards an overfished state, while LATF is fully exploited as documented by [25,27]. The estimated MSY for bonga shad from CMSY++ and BSM was lower than the most recent annual catch (147.9 × 10^3^ t·yr^−1^), indicating that recent removals likely exceed sustainable production. In contrast, lesser African threadfin’s MSY estimates from CMSY++ and BSM were close to recent catch levels (10.2 × 10^3^ t·yr^−1^). Previous studies [60] have noted that when MSY approximates recent catch levels, the stock is generally considered fully exploited [59,61]. Our results indicate that, while AMSY and CMSY++ classified bonga shad’s stock as fully exploited, the BSM suggested overfishing conditions. Collectively, these results indicate that bonga is at least fully exploited and may be transitioning toward an overfished state if current fishing pressure persists. For LATF, although AMSY indicated an overfished status, both CMSY++ and BSM classified LATF’s stock as fully exploited. Overall, this suggests that the stock is primarily fully exploited but operating near its sustainable limit, with limited buffer against additional increases in fishing pressure. Accordingly, our results suggest that bonga shad is transitioning from a fully exploited condition toward overfishing, while LATF remains fully exploited, consistent with earlier findings [25,27].

Under such circumstances, a TAC below MSY or a reduced fishing mortality (F < F_MSY_) is recommended to promote stock rebuilding and reduce the risk of further depletion, particularly given uncertainties associated with data-limited assessments.

This study analyzed the model’s fitting ability by checking the distribution of residual patterns as well as the distribution of posterior parameter estimates. Residual diagnostics based on run tests of the log-CPUE residuals from the Bayesian Schaefer Model (BSM) showed no evidence of systematic bias, suggesting an adequate model fit in compliance with the assumption of randomly distributed residuals [18]. Ref. [62] suggested that, when the prior perception of stock status deviates from the available data, prior knowledge would be adjusted by CMSY++ and its BSM analysis. Overall patterns align with expectations for data-limited, relative-abundant-based assessments. The absence of strong autocorrelation supports the reliability of the BSM frameworks and derived reference points. The retrospective analysis is a widely applied diagnostic tool in fisheries science for evaluating the stability, reliability, and temporal coherence of stock assessment models [63,64]. Examining the sensitivity of model outputs to the sequential removal of terminal-year data, it provides insight into whether observed trends reflect genuine stock dynamics of data availability and model structure. Ideally, a robust model should remain responsive to new information while maintaining stable trajectories through time [65]. In this study, retrospective analyses based on AMSY and the Bayesian Schaefer Model (BSM) were consistent for *Ethmalosa fimbriata* and *Galeoides decadactylus*’ stocks. For bonga shad, both models indicated increasing fishing mortality (F/F_MSY_) and declining biomass relative to MSY (B/B_MSY_) over time. AMSY displayed a moderate retrospective bias, with earlier peels underestimating exploitation and overestimating biomass, followed by systematic upward revisions of F/F_MSY_ and downward revisions of B/B_MSY_ as recent data were incorporated. Such behavior is characteristic of data-limited surplus production models and has been widely documented in small pelagic fisheries [27,46]. In contrast, BSM exhibited tighter convergence across peels, reflecting the stabilizing influence of its Bayesian state-space formulation [66]. while still converging toward the same qualitative conclusion of declining stock condition. Similar trends for *E. fimbriata* and other small pelagic stocks in the Gulf of Guinea have been linked to sustained fishing pressure and reduced productivity [67,68].

For *Galeoides decadactylus*, retrospective patterns from AMSY and BSM were consistent. AMSY truncations systematically revised terminal-year estimates toward higher F/F_MSY_ and lower B/B_MSY_, a pattern widely reported in relative abundance-based assessments where recent periods of intensified exploitation dominate model trajectories [16,69]. Comparable retrospective bias has been observed in data-limited assessments of West African fisheries, where the abundance index is influenced by technological creep, effort concentration, and changing fishing strategies [46,61]. The stronger convergence toward overfished conditions aligns with previous research that reported an increase in fishing capacity and effort concentration [70,71]. BSM consistently showed tighter grouping of retrospective peels, particularly for biomass trajectories, consistent with previous findings that Bayesian surplus production models dampen retrospective volatility through explicit process-error estimation and probabilistic constraints [31,72]. However, BSM revised terminal-year biomass upward and fishing mortality downward as additional data were incorporated, a pattern increasingly recognized in Bayesian assessments of fully exploited, data-limited fisheries [73,74]. Overall, the convergence of AMSY and BSM across species reflects a real intensification of fishing pressure. The concordance with findings from other West African and global small-scale fisheries strengthens confidence in the assessment results and supports precautionary management advice [16,47,50]. According to [62], there is a 94% correlation between CMSY++ predictions and BSM results in comparison with other stocks from different fisheries. The stocks of *E. fimbriata* and *G. decadactylus* are conventionally treated by CECAF as shared regional stocks in the Gulf of Guinea. However, recent studies suggest that small pelagic and coastal species may exhibit partial population structuring [75,76], a phenomenon that may occur across the Gulf of Guinea coastline. Consequently, MSY values should not be interpreted as precise production targets but rather as precautionary reference points. In this study, the relatively wide uncertainty ranges and variation in MSY estimates across models highlight the limitations of the available data and reinforce the need to interpret these values cautiously when informing management decisions. The estimated reference points should be interpreted as indicators of a broad regional production unit rather than a strictly discrete biological stock. Accordingly, management advice derived from these values should be applied cautiously and complemented by improved spatial monitoring and country-specific data collection to reduce uncertainty. The relatively wide confidence intervals observed for MSY and stock status metrics reflect inherent limitations of catch-only and catch-plus-index assessment approaches. Uncertainty arises primarily from three sources: (i) dependence on prior assumptions for intrinsic growth rate (r) and carrying capacity (K), (ii) the limited information content of catch data, which requires indirect reconstruction of biomass dynamics, and (iii) potential violations of the assumption that CPUE remains proportional to abundance through time. Similar magnitudes of variability are widely reported for CMSY-type methods, where MSY confidence intervals frequently span ±40–80% or more of the median estimate [16,77]. Consequently, MSY values should not be interpreted as precise production targets but rather as precautionary reference points for evaluating relative exploitation status.

In data-limited fisheries such as those of the Gulf of Guinea, the relationship between catch and CPUE is not always expected to be positively correlated, particularly in small pelagic fisheries. Increases in total catch may reflect increased fishing effort or improved fishing efficiency rather than increases in stock abundance. In such cases, CPUE may decline even as catches increase, resulting in weak or negative correlations between the two variables.

Additionally, species such as *Ethmalosa fimbriata* exhibit schooling behavior, which can lead to hyperstability in catch rates, whereby CPUE remains relatively stable or declines slowly despite reductions in stock biomass. These dynamics can complicate the interpretation of CPUE as a direct proxy for abundance.

Although standardized CPUE indices were used to mitigate the influence of effort and environmental variability, some residual bias may remain. Therefore, the abundance indices should be interpreted as relative indicators of biomass trends, and results should be considered within a precautionary management framework.

Also, risk-averse strategies are therefore recommended, whereby exploitation rates are maintained below the lower confidence bound of MSY or biomass is kept safely above B_MSY_. Despite these uncertainties, catch-based models remain valuable because they integrate life-history information, historical removals, and available abundance signals within a coherent population-dynamics framework, providing probabilistic estimates of stock condition and explicit measures of risk. Their primary role is therefore to inform broad management direction rather than to define exact allowable catches. Precautionary measures such as effort controls or gradual catch reductions toward sustainable levels [78] may help stabilize and rebuild stocks in the Gulf of Guinea. In addition to TAC adjustments, improving monitoring systems is critical. Priority actions include the standardization and continuous collection of CPUE data across fleets, enhanced biological sampling (e.g., length-frequency and maturity data), and improved reporting of catch and effort. Strengthening these data streams will reduce uncertainty in future assessments and support more adaptive and evidence-based fisheries management in the region.

## 5. Conclusions

This study applied reliable, complementary data-limited assessment approaches. The stock parameter estimates for bonga shad based on AMSY and CMSY++ showed the stock is fully exploited, while BSM suggests an overfished stock status in the last year. While CPUE-based models can provide important information on stock dynamics, their reliability depends strongly on the quality, consistency, and representativeness of the underlying abundance indices. In the present study, uncertainties associated with the available CPUE series may have influenced biomass estimates, highlighting the importance of careful interpretation and improved data quality in data-limited stock assessments.

In contrast, for lesser African threadfin (LATF), the stock status remains uncertain; however, catch-only model results suggest that biomass may be above B_MSY_ and fishing pressure below FMSY. Given the inconsistency in the CPUE series, these results should be interpreted cautiously, and improved data collection is required for a more robust assessment. The estimated MSY values for bonga shad were below the last year’s catch, indicating a risk of long-term stock depletion, whereas MSY estimates for LATF were close to the last year’s catch according to the CMSY++ model. Therefore, there is an urgent need for species-specific management measures in the fisheries in the region.

The findings of the present study suggest that immediate precautionary management measures are necessary to ensure the sustainability of *Ethmalosa fimbriata* and *Galeoides decadactylus* fisheries in the Central Gulf of Guinea. From a management perspective, the observed decline in biomass and increasing exploitation pressure, particularly for *E. fimbriata*, highlight the need for precautionary harvest strategies in the Central Gulf of Guinea. Recommended measures include reducing fishing effort through limits on fishing days and active vessels, particularly within heavily exploited artisanal and industrial fleets, and the implementation of seasonal closures during peak spawning periods to enhance stock recovery and recruitment success. Strengthening mesh-size regulations and limiting juvenile catches are also essential, especially for *E. fimbriata*, which is highly vulnerable to recruitment overfishing, implementing catch controls within estimated MSY ranges, and enforcing seasonal closures during key spawning periods to support stock recovery. Restrictions on non-selective fishing gears targeting juvenile fish may further improve stock sustainability. Given the strong dependence of coastal communities on artisanal fisheries for income, employment, and food security, management measures should be implemented progressively within an adaptive co-management framework that balances resource conservation with socioeconomic sustainability. Given the transboundary nature of these fisheries, regional cooperation through CECAF and stronger control of industrial fishing activities will be critical for promoting long-term ecosystem sustainability and effective fisheries governance in the Gulf of Guinea. Future efforts should prioritize improving the quality and continuity of catch and CPUE data, integrating environmental drivers where possible, strengthening regional collaboration in data collection, and assessing other fish stocks to ensure the sustainability of the fishery resources in the CGG.

## Figures and Tables

**Table 1:** r and B/k (relative biomass) prior ranges used and inputs used in the Markov Chains. Monte Carlo method (CMSY++ and BSM) analysis.

Stock	Resilience	r.low	r.hi	Start B/k Low	Start B/K High	Final B/k	Intermediate B/k
Bonga shad	High	0.15	0.34	0.2	0.8	Not specified	Not specified
LATF	Medium	0.32	0.73	0.2	0.8	Not specified	Not specified

**Table 2:** Estimated carrying capacity bounds ($k_{low}$ and $k_{high}$) for *Ethmalosa fimbriata* and *Galeoides decadactylus* based on maximum catch and resilience priors.

Species	r Range	Max Catch (t)	k_Low (t)	k_High (t)	Equation Used	Rationale
Bonga shad	0.15–0.34	152,805	4.49 × 10^5^	1.22 × 10^7^	Equation (3)	Declining CPUE
LATF	0.32–0.73	11,282	1.55 × 10^4^	4.23 × 10^5^	Equation (3)	Declining CPUE

**Table 3:** Biological reference points (Median with lower and Upper limits of 95% confident intervals (CI)) for bonga shad estimated using AMSY, CMSY++, and BSM in the Central Gulf of Guinea.

Model	MSY (10^3^ t yr^−1^) (95% CI)	F_MSY_ (yr^−1^)	B_MSY_ (10^3^ t)	B/B_MSY_ (Last)	F/F_MSY_ (Last)	Stock Status
AMSY	–	0.17	–	0.77	0.83	Fully exploited
CMSY++	126 (90.3–201)	0.085	1466.5	1.08	1.03	Fully exploited
BSM	95.5 (73.5–134)	0.10	915	0.87	1.77	Overfished

**Table 4:** Biological reference points with 95% confident intervals for LATF estimated using AMSY, CMSY++, and BSM in the Central Gulf of Guinea.

Model	MSY (10^3^ t yr^−1^) (95% CI)	F_MSY_ (yr^−1^)	B_MSY_ (10^3^ t)	B/B_MSY_ (Last)	F/F_MSY_ (Last)	Stock Status
AMSY	–	0.245	–	0.50	1.20	Overfished
CMSY++	9.1 (6.7–13.2)	0.165	54	1.08	1.05	Fully exploited
BSM	13.4 (9.2–20.4)	0.178	75.2	0.91	0.85	Fully exploited

## Data Availability

The data is available on the public repository online link at https://drive.google.com/file/d/1ofvk12zmfJbttKHJzAfJNq73ztyPRGCE/view?usp=drive_link, accessed on 29 March 2026.

## Supplementary Materials

The following supporting information can be downloaded at: https://www.mdpi.com/article/10.3390/biology15120978/s1, Figure S1: Analyses of viable r-k pairs plots from AMSY, CMSY++ and BSM with 95% confident intervals (shaded area) for bonga shad in the CGG; Figure S2: Analyses of viable r-k pairs plots from AMSY, CMSY++ and BSM with 95% confident intervals (shaded area) for LATF in CGG, Figure S3: Prior (light color) and posterior (dark color) distributions of parameters for bonga shad based on CMSY++ and BSM in the CGG, Figure S4: Prior (light color) and posterior (dark color) distributions of parameters for LATF based on CMSY++ and BSM, Table S1: Life-history parameters and estimation procedures used in AMSY, CMSY++ and BSM analyses for Bonga shad and Lesser African threadfin; Table S2: Catch time series for Bonga shad (A) and Lesser African threadfin(B) used in AMSY, CMSY++ and BSM analyses, Table S3a: Standardized relative abundance index for Bonga shad, Table S3b: Standardized and scaled relative abundance index for Bonga shad used in AMSY, CMSY++ and BSM analyses, Table S4a: Standardized relative abundance index for Lesser African threadfin, Table S4b: Standardized and scaled relative abundance index for Lesser African threadfin used in AMSY, CMSY++ and BSM analyses, Table S5a: Biological reference points with 95% confident intervals for bonga shad estimated using AMSY, CMSY++ and BSM models in the Central Gulf of Guinea, Table S5b: Biological reference points with 95% confident intervals for LATF estimated using AMSY, CMSY++ and BSM in the Central Gulf of Guinea.
