# Supplementary material for: Data-Limited Stock Status Assessment of Bonga Shad, Ethmalosa fimbriata (Bowdich, 1825) and Lesser African Threadfin, Galeoides decadactylus (Bloch, 1795) in the Central Gulf of Guinea
Source: Biology (Basel). 2026 Jun 22;15(12):978. doi: 10.3390/biology15120978 (PMC13295871; doi:10.3390/biology15120978)
Supplement: Supplementary file 1 [file biology-15-00978-s001.zip › biology-4256920-supplementary.pdf]

SUPPLEMENTARY MATERIALS:

Title: Data-Limited Stock Status Assessment of Bonga Shad, *Ethmalosa fimbriata* (Bowdich, 1825) and Lesser African Threadfin, *Galeoides decadactylus* (Bloch, 1795) in the Central Gulf of Guinea

Supplementary table (ST)

Table S1: Life-history parameters and estimation procedures used in AMSY, CMSY++ and BSM analyses for Bonga shad and Lesser African threadfin

Parameter	Bonga shad	Lesser African threadfin	Estimation method / equation	Source
Asymptotic length (L_∞, cm)	29.1	36.8	von Bertalanffy growth function fitted to length-frequency data (ELEFAN)	Estimated using TropFishR
Growth coefficient (K, yr^{-1})	0.62	0.29	von Bertalanffy growth function	Estimated using TropFishR
Natural mortality (M, yr^{-1})	0.8–1.1 (median 0.92)	0.4–0.6 (median 0.51)	Pauly empirical model: $\log_{10}(M) = -0.0066 - 0.279\log_{10}L\infty + 0.6543\log_{10}K + 0.463\log_{10}T$	[26]
Length-weight (a, b)	a = 0.015, b = 2.87	a = 0.017, b = 2.85	Nonlinear regression of $W = aL^b$ from sampled fish	Estimated using TropFishR
L_{m50}, mm	227	218	Logistic maturity ogive fitted to length data	Estimated using TropFishR maturity module
L_{m95}, mm	252	289	Logistic maturity ogive	Estimated using TropFishR
Maximum age (Amax, yr)	4-5	3-4	Approx. Amax ≈ 3/K	Beverton-Holt life-history relationship
Selectivity (SL50, SL95, mm)	253, 303	205, 300	Logistic gear selectivity curve fitted to catch-at-length	Estimated using TropFishR
Catchability (q)	Estimated internally	Estimated internally	Derived during AMSY/BSM likelihood optimization	Model-estimated
Process error (σprocess)	0.08-0.20	0.08-0.20	Log-normal prior	CMSY/BSM defaults
Observation error (σobs)	0.15	0.15	Fixed log-normal observation variance	CMSY/BSM defaults
Initial depletion (B_{start}/K)	0.0001-1.5	0.0001-1.5	Log-normal prior range	CMSY/BSM
Final depletion (B_{final}/K)	0.2 (prior mean)	0.2	Informed prior based on recent exploitation	CMSY/BSM

Table S2: Catch time series for Bonga shad (A) and Lesser African threadfin(B) used in AMSY, CMSY++ and BSM analyses

(A)

Year	Catch
1990	2592
1991	3424
1992	1759
1993	1169
1994	1178
1995	1932
1996	4004
1997	6474
1998	2356
1999	3034
2000	4756
2001	5593
2002	4953
2003	6679
2004	4926
2005	3938
2006	6339
2007	4756
2008	5540
2009	5267
2010	5016
2011	4744
2012	6426
2013	7140
2014	7322
2015	7431
2016	8441
2017	11142
2018	11282
2019	10405
2020	9877
2021	10244
2022	10061
2023	10153

(B)

Year	Catch
1990	30883
1991	33816
1992	55946
1993	41670
1994	45423
1995	41004
1996	32647
1997	57974
1998	55572
1999	45063
2000	43935
2001	46287
2002	48722
2003	48526
2004	50459
2005	65395
2006	70521
2007	67937
2008	73678
2009	79678
2010	85273
2011	88856
2012	94261
2013	100952
2014	106553
2015	111936
2016	116849
2017	146596
2018	151198
2019	152805
2020	144580
2021	149041
2022	146811
2023	147926

Table S3a: Standardized relative abundance index for Bonga shad

**Year**	Standardized relative abundance for bonga shad from artisanal efforts in Cam
1994	0.06383869
1995	0.12328323
1996	0.02694975
1997	0.00513371
1998	0.01043693
1999	0.11822021
2000	0.37141526
2001	0.11841474
2002	0.04044564
2003	0.05717184
2004	0.00273913
2005	0.00129426
2006	0.00368983
2007	0.00647422
2008	0.17148735

(Source:[29])

Table S3b: Standardized and scaled relative abundance index for Bonga shad used in AMSY, CMSY++ and BSM analyses

**Year**	**Standardized and Scaled* Relative abundance index for Bonga shad**
1994	**0.08511825**
1995	**0.16437764**
1996	**0.03593299**
1997	**0.00684495**
1998	**0.0139159**
1999	**0.15762695**
2000	**0.49522034**
2001	**0.15788632**
2002	**0.05392752**
2003	**0.07622911**
2004	**0.00365217**
2005	**0.00172568**
2006	**0.00491977**
2007	**0.00863229**
2008	**0.2286498**

Scaled*= relative value/mean

Table S4a: Standardized relative abundance index for Lesser African threadfin

Year	Standardized abundance index for LATF from artisanal efforts in Cameroon 1	Standardized abundance index for LATF from industrial fishing efforts in Cameroon 2	Standardized abundance index for LATF from artisanal efforts in Nigeria 1	Standardized abundance index for LATF from industrial fishing efforts in Nigeria 2
1990	101.393028	5.66351393		0.896281125
1991	107.041947	8.75284523		2.203865487
1992	74.6020104	1.63783278		10.45174142
1993	66.6481962	4.56462357	98	13.3742431
1994	60.5999788	3.24607527	271	35.80384801
1995	70.8118051	4.04447103	332	39.17443942
1996	54.6367588	8.1909013	459	52.21834923
1997	58.2390038	3.22335831	474	32.18716172
1998	64.1700805	6.16336876	876	24.6952504
1999	55.6639537	4.15715926	981	37.20332195
2000	29.7401037	2.11213966	1068	29.87334197
2001	23.5793377	2.31792293	902	20.83570066
2002	18.8106648	2.11658502	832	18.45626172
2003	17.4270271	1.58838448	721	17.43534817
2004	22.6439524	1.8992454	629	10.28401956
2005	35.210936	3.2765897	77	10.58305461
2006	37.3534075	0.3047877	53	11.78363802
2007	43.832324	3.66819721	27	21.80488296
2008	9.764484	2.54373677	28	14.6719747
2009	2.39467222	0.90965243	22	13.69184524
2010	5.36553353	2.17266051	44	17.62044717
2011	3.15569667	0.6710185	112	19.14880362
2012	0.53789703	0.02454228	200	20.06911652
2013	4.56097473	1.678651	140	21.94034886
2014	1.26681805	0.21266104	207	23
2015	0.7479609	0.10607145	300	22
2016	4.58391955	2.01625351	315	28

(Source :[28])

Table S4b: Standardized and scaled relative abundance index for Lesser African threadfin used in AMSY, CMSY++ and BSM analyses

Year	Standardized and scaled* Relative abundance index in Cameroon 1	Standardized and scaled* Relative abundance index Cameroon 2	Standardized and scaled* Relative abundance index in Nigeria 1	**Standardized and** scaled* Relative abundance index in Nigeria 2	**Average of Standardized and scaled* relative abundance index for LATF**
1990	2.80	2.04		0.043	**1.2315**
1991	2.96	3.15		0.107	**1.581**
1992	2.06	0.59		0.505	**0.915**
1993	1.84	1.64	0.26	0.647	**1.09675**
1994	1.67	1.17	0.71	1.732	**1.3205**
1995	1.96	1.45	0.86	1.894	**1.541**
1996	1.51	2.95	1.19	2.525	**2.04375**
1997	1.61	1.16	1.23	1.556	**1.389**
1998	1.77	2.22	2.28	1.194	**1.866**
1999	1.54	1.50	2.55	1.799	**1.84725**
2000	0.82	0.76	2.78	1.445	**1.45125**
2001	0.65	0.83	2.35	1.007	**1.20925**
2002	0.52	0.76	2.17	0.892	**1.0855**
2003	0.48	0.57	1.88	0.843	**0.94325**
2004	0.63	0.68	1.64	0.497	**0.86175**
2005	0.97	1.18	0.20	0.512	**0.7155**
2006	1.03	0.11	0.14	0.570	**0.4625**
2007	1.21	1.32	0.07	1.055	**0.91375**
2008	0.27	0.91	0.07	0.709	**0.48975**
2009	0.07	0.33	0.06	0.662	**0.2805**
2010	0.15	0.78	0.11	0.852	**0.473**
2011	0.09	0.24	0.29	0.926	**0.3865**
2012	0.01	0.01	0.52	0.971	**0.37775**
2013	0.13	0.60	0.36	1.061	**0.53775**
2014	0.04	0.08	0.54	1.112	**0.443**
2015	0.02	0.04	0.78	1.064	**0.476**
2016	0.13	0.72	0.82	1.354	**0.756**

Scaled*= relative value/mean

Table S5a: **Biological reference points with 95% confident intervals for bonga shad estimated using AMSY, CMSY++ and BSM models in the Central Gulf of Guinea.**

Model	r (yr^{-1}) (95% CI)	k (10^3 t) (95% CI)	MSY (10^3 t yr^{-1}) (95% CI)	Fmsy (yr^{-1}) (95% CI)	F last (yr^{-1}) (95% CI)	Bmsy (10^3 t) (95% CI)	B/K (95% CI)	B last (10^3 t) (95% CI)	B last/Bmsy (95% CI)	F last/Fmsy (95% CI)	Stock status
**AMSY**	0.35 (0.23,0.52)	–	–	0.17 (0.12-0.26)	–	–	0.39 (0.21,0.7)	–	0.77 (0.42-1.4)	0.83 (0.05-2.64)	**Fully exploited**
**CMSY++**	0.17 (0.12,0.22)	2933 (2120-5125)	126 (90.3-201)	0.085 (0.06-0.11)	–	1466.5 (1060-2562.5)	0.55 (0.31-0.73)	–		1.03 (0.53-2.43)	**Fully exploited**
**BSM**	0.209 (0.15-0.29)	1829 (1214-3032)	95.5 (734.5-134)	0.10 (0.08-0.15)	0.19 (0.08-0.44)	915 (607-1516)	0.44 (0.24-0.65)	792 (350-1691)	0.87 (0.48-1.29)	1.77 (0.87-4.08)	**Overfished**

Table S5b: **Biological reference points with 95% confident intervals for LATF estimated using AMSY, CMSY++ and BSM in the Central Gulf of Guinea.**

Model	r (yr^{-1}) (95% CI)	k (10^3 t) (95% CI)	MSY (10^3 t yr^{-1}) (95% CI)	Fmsy (yr^{-1}) (95% CI)	F last (yr^{-1}) (95% CI)	Bmsy (10^3 t) (95% CI)	B/K (95% CI)	B last (10^3 t) (95% CI)	B last/Bmsy (95% CI)	F last/Fmsy (95% CI)	Stock status
**AMSY**	0.49 (0.34–0.69)	–	–	0.245 (0.17–0.345)	–	–	0.25 (0.135–0.43)	–	0.50 (0.27–0.86)	1.20 (0.14–2.90)	**Overfished**
**CMSY++**	0.33 (0.23–0.43)	108 (81–177)	9.1 (6.7–13.2)	0.165 (0.115-0.215)	–	54 (40.5–88.5)	0.54 (0.34–0.71)	–	1.08* (0.68–1.43)	1.05 (0.55–2.12)	**Fully exploited**
**BSM**	0.356 (0.244–0.518)	150 (90–265)	13.4 (9.2–20.4)	0.178 (0.12–0.26)	0.15 (0.07–0.34)	75.2 (45–133)	0.45 (0.242–0.665)	66.9 (30.6–133)	0.905 (0.485–1.33)	0.85 (0.43–1.86)	**Fully exploited**

*** CMSY B/Bmsy derived from B/K using Schaefer relationship (B/Bmsy = 2B/K)**

Supplementary Figures (S): Analyses of viable r-k pairs plots

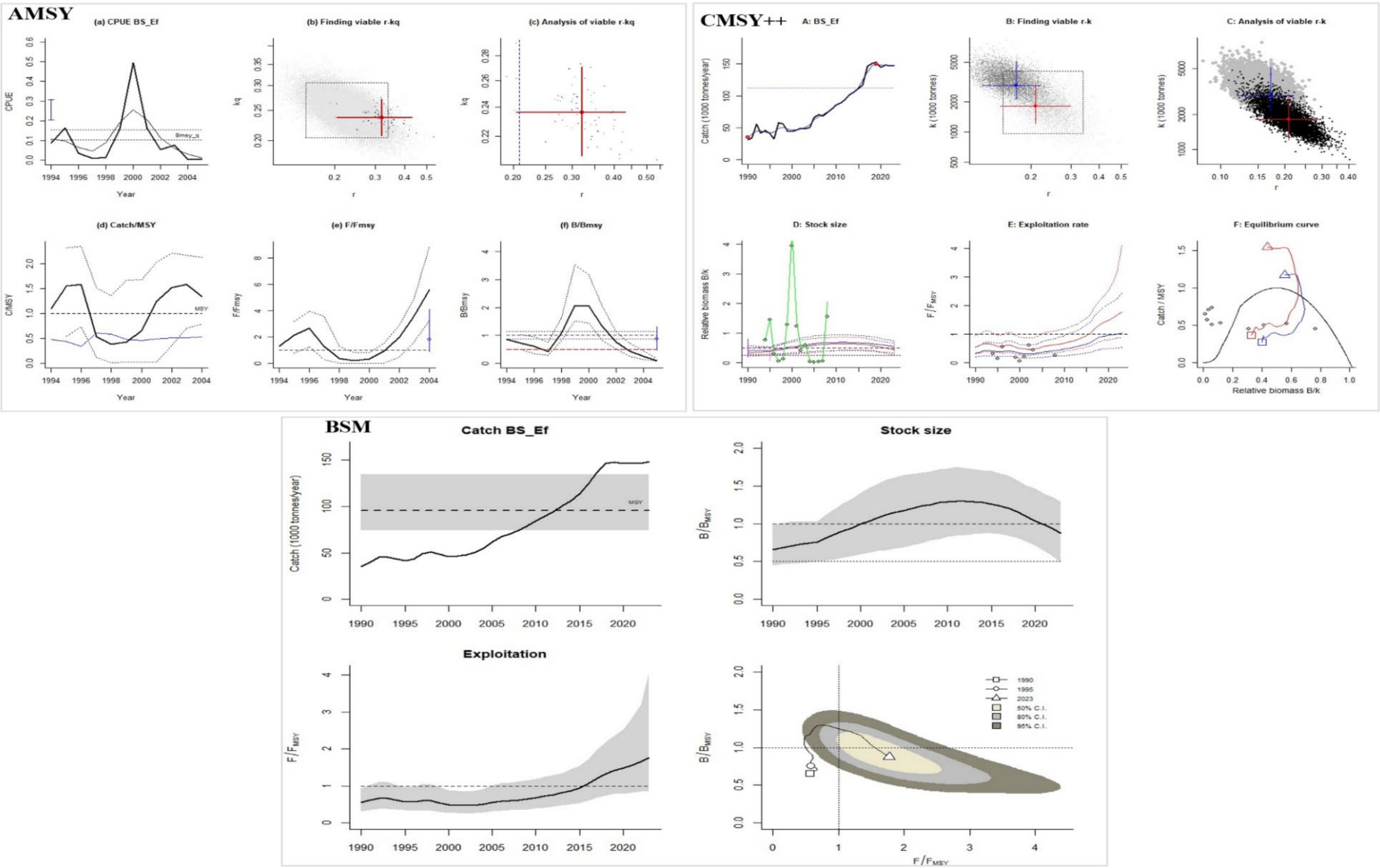

Figure S1: Analyses of viable r-k pairs plots from AMSY, CMSY++ and BSM with 95% confident intervals (shaded area) for bonga shad in the CGG

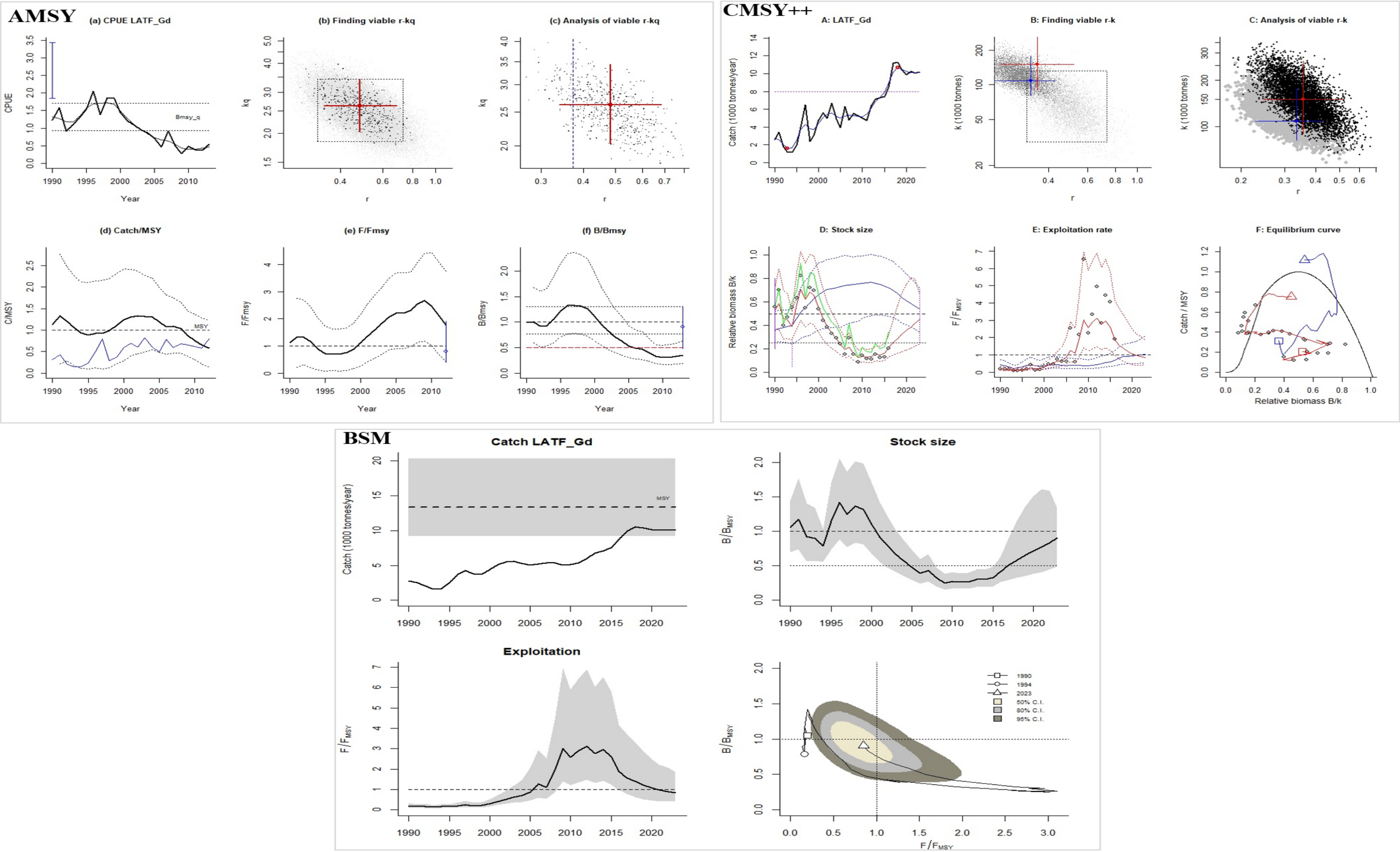

Figure S2: Analyses of viable r-k pairs plots from AMSY, CMSY++ and BSM with 95% confident intervals (shaded area) for LATF in CGG

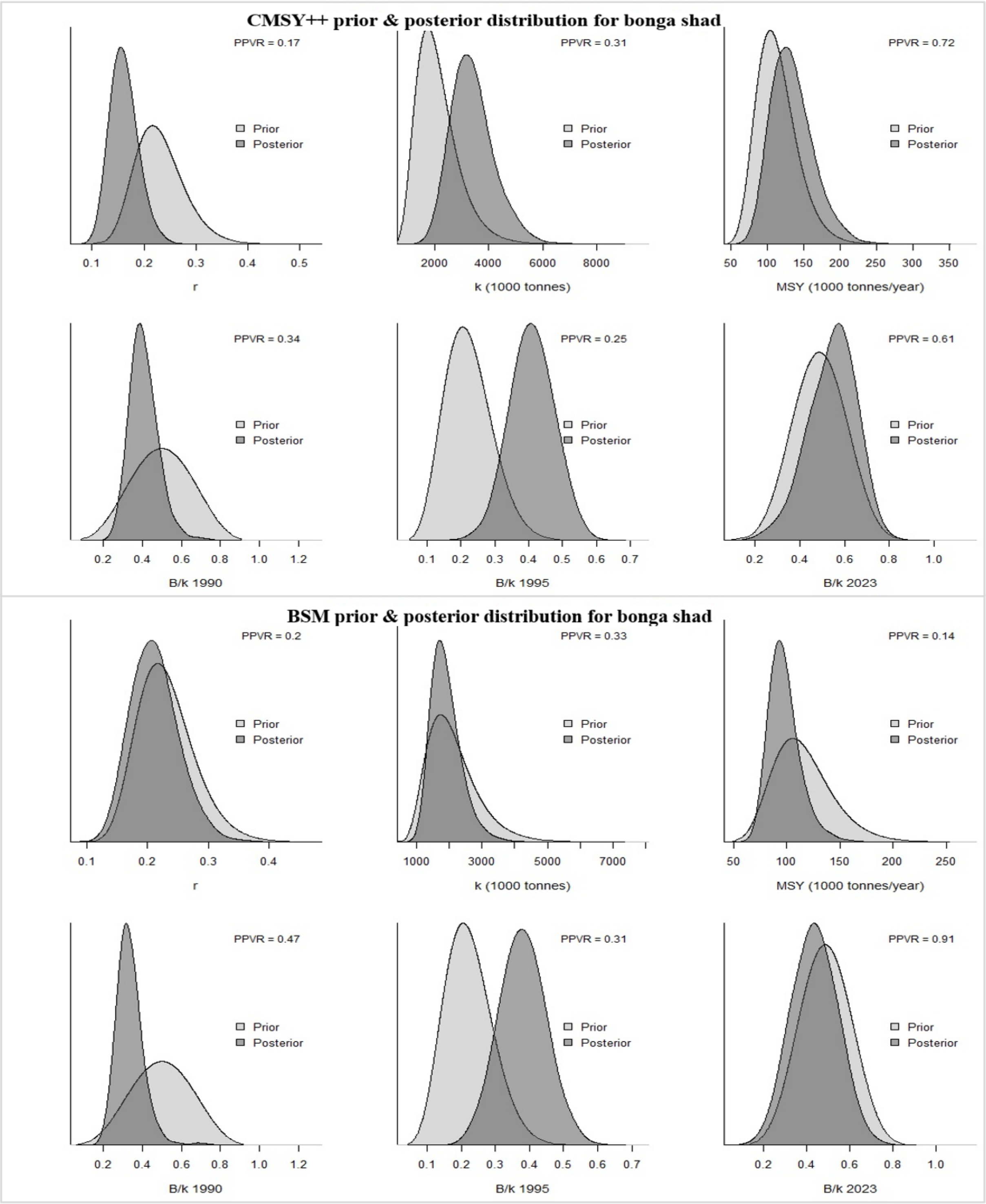

Figure S3: Prior (light color) and posterior (dark color) distributions of parameters for bonga shad based on CMSY++ and BSM in the CGG

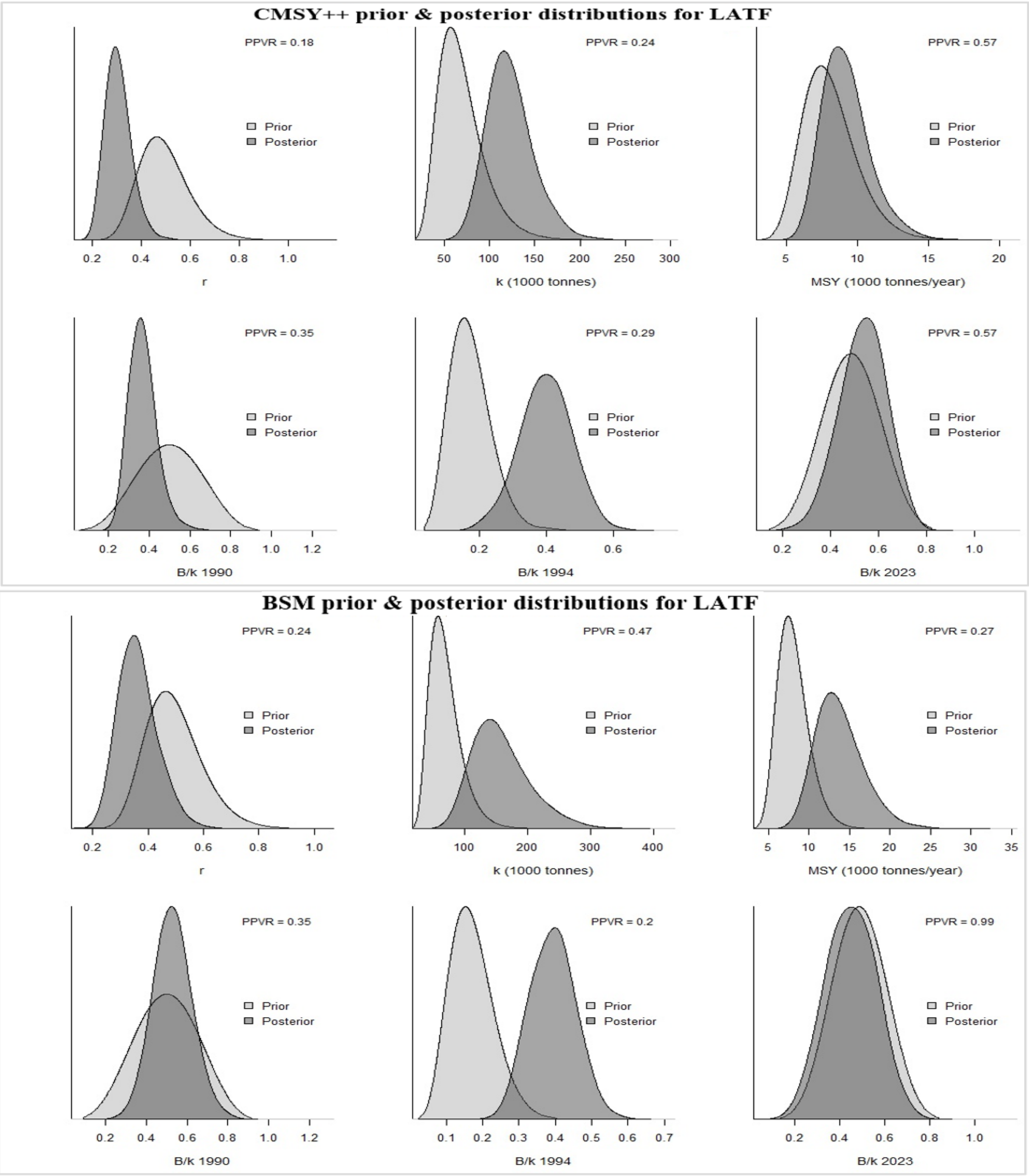

Figure S4: Prior (light color) and posterior (dark color) distributions of parameters for LATF based on CMSY++ and BSM
